# IgG4-Related Kidney Disease: Report of a Case Presenting as a Renal Mass

**DOI:** 10.1155/2017/9690218

**Published:** 2017-08-22

**Authors:** Daniele Bianchi, Luca Topazio, Gabriele Gaziev, Valerio Iacovelli, Pierluigi Bove, Alessandro Mauriello, Enrico Finazzi Agrò

**Affiliations:** ^1^Department of Urology, Policlinico Tor Vergata, Rome, Italy; ^2^Anatomic Pathology, Department of Experimental Medicine and Surgery, Tor Vergata University, Rome, Italy

## Abstract

IgG4-related disease (IgG4-RD) is a nosological entity defined as a chronic immune-mediated fibro-inflammatory condition characterized by a tendency to form tumefactive, tissue-destructive lesions or by organ failure. Urologic involvement in IgG4-RD has been described in some short series of patients and in isolated case reports, most often involving the kidneys in so-called IgG4-related kidney disease (IgG4-RKD). The disease can occasionally mimic malignancies and is at risk of being misdiagnosed due to its rarity. We report the case of a 56-year-old man presenting with a right renal mass suspected of being malignant. Laboratory tests showed normal creatinine levels, a high erythrocyte sedimentation rate, and high levels of C-reactive protein and microalbuminuria. The patient underwent radical right nephroureterectomy and histopathologic examination revealed features proving IgG4-RKD. He was therefore referred to immunologists. Typical clinical presentation of IgG4-RKD includes altered renal function with inconstant or no radiologic findings. Conversely, in the case we presented, a single nodule was detected upon imaging evaluation, thus mimicking malignancy. This raises the issue of a proper differential diagnosis. A multidisciplinary approach can be useful, although in clinical practice the selection of patients suspected of having IgG4-RKD is critical in the cases presenting with a renal mass that mimics malignancy.

## 1. Introduction

IgG4-related disease (IgG4-RD) is a recent nosological entity defined as a chronic immune-mediated fibro-inflammatory condition [1] characterized by tumefactive, tissue-destructive lesions or by organ failure [2]. IgG4-RD potentially involves nearly every anatomic site [3, 4], occasionally including urologic structures, as described in some short series of patients and in isolated case reports [5]. Typical histopathologic features are lymphoplasmacytic infiltrate rich in IgG4 plasma cells, obliterative phlebitis, and storiform fibrosis, while laboratory tests may reveal an inconstantly elevated serum IgG4 concentration [6].

IgG4-related kidney disease (IgG4-RKD) is the most common among urologic manifestations of IgG4-RD, usually presenting in the form of tubulointerstitial nephritis (TIN) [7–9], although some cases of membranous glomerulonephritis (MGN) have been described [9–11]. IgG4-RKD presenting as a solid renal mass has rarely been described [5], and conventional imaging—including ultrasound scans, computed tomography (CT), and magnetic resonance imaging (MRI)—has proven to be of limited usefulness in determining IgG4-RD [12].

## 2. Case Report

**Figure 1:**
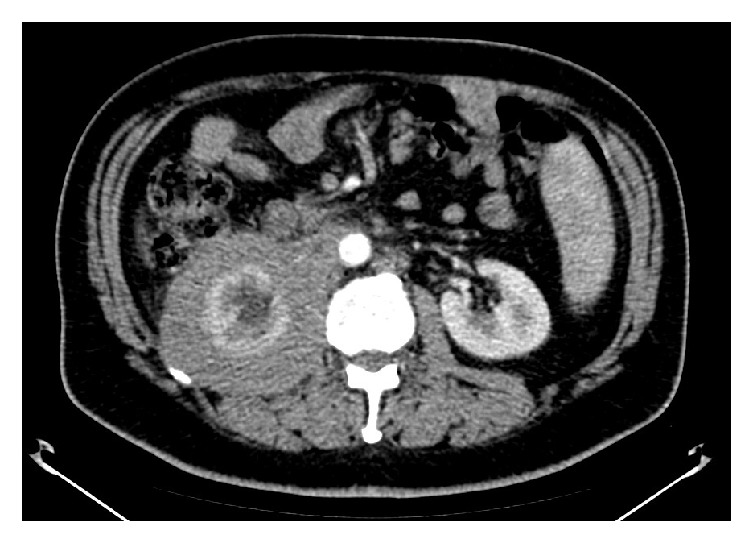
Preoperative computed tomography (CT) scan showing a right renal mass.

**Figure 2:**
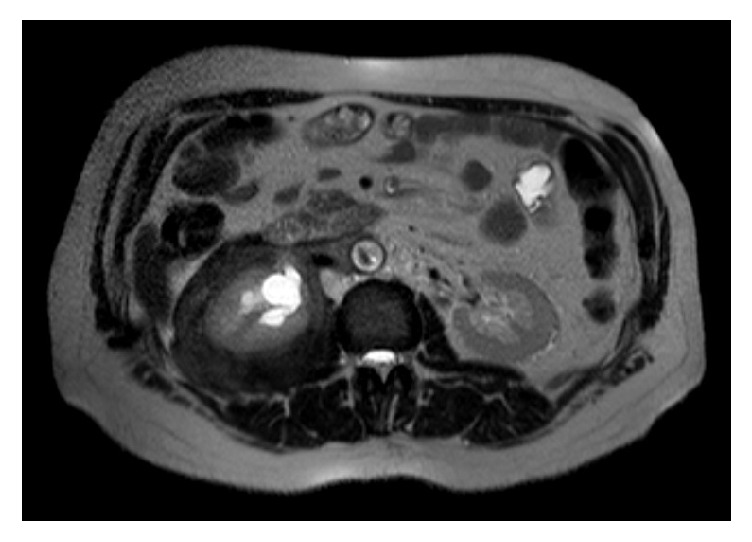
Preoperative magnetic resonance imaging (MRI) scan showing a right renal mass.

We report the case of a 56-year-old Caucasian man presenting with a right renal mass (12 × 9 × 8 cm) revealed by CT (Figure 1) and MRI (Figure 2). The mass was localized at the upper pole of the right kidney, extending to the renal fascia towards the caval vein and iliac vessels and involving the vascular hilum. The patient complained of moderate abdominal pain over the last weeks prior to urologic consultation.

Laboratory tests performed within four months prior to urologic evaluation showed creatinine level of 1.10 mg/dL, a erythrocyte sedimentation rate up to 120 mm/h, C-reactive protein levels ranging from 65 to 70 mg/L, and microalbuminuria levels of 82.5 mg/g. These high levels drove the decision for abdominal imaging. Comorbidities included mellitus diabetes type 2. No further relevant aspects were recorded in the patient's previous history.

Due to the suspicion of malignancy, the patient underwent open-surgery radical right nephroureterectomy, which was complicated by firm adhesions between the kidney, its fascia, and surrounding structures so that the posterior renal fascia could not be excised. The postoperative period was uneventful.

**Figure 3:**
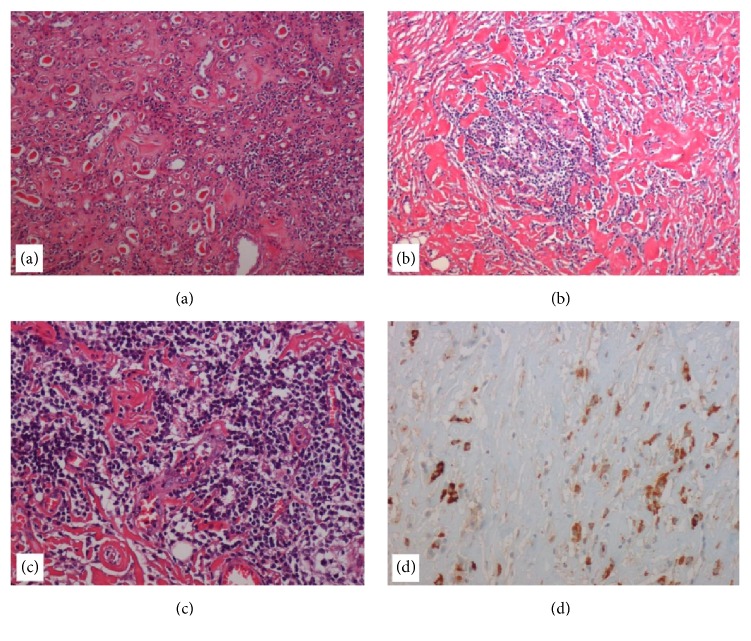
Histological findings. (a) Histological section of kidney parenchyma showing a chronic pyelonephritis characterized by the presence of interstitial fibrosis, inflammatory infiltrate of lymphocytes and plasma cells, and atrophy and dilatation of tubules that contain pink casts (hematoxylin-eosin (H-E), 10x). (b) The fibroadipose tissue of the kidney capsule typically shows the histological findings of IgG4-related disease, characterized by an irregularly whorled pattern of fibrosis (storiform fibrosis) associated with inflammatory infiltrate (H-E, 10x). (c) A vein obliterated by a transmural inflammatory cell infiltration (obliterative phlebitis) (H-E, 20x). (d) Immunostaining with anti-IgG4 antibody shows the presence of numerous positive cells (×20).

Histopathologic examination showed storiform fibrosis with lymphoplasmacytic infiltrate made up of B and T cells (CD20+, CD3+), as well as focal obliterative phlebitis. The IgG4+/IgG ratio was 70% (Figure 3). Given the histologic features proving IgG4-RKD, the patient was referred to immunologists and underwent blood tests, including total IgG (1150 mg/dL) and IgG4 (69 mg/dL). Further rheumatologic tests (rheumatoid factor, anticardiolipin antibody, cANCA, pANCA, anti-Jo-1 antibody, complement factors C3 and C4, a Waaler Rose test, and Sm-RNP) were negative.

The patient was prescribed 5 mg prednisone daily and 50 mg azathioprine twice daily for the first five months after surgery and experienced dramatic pain improvement. Afterwards, a drug maintenance regimen of 50 mg azathioprine twice daily was prescribed by immunologists. Currently, at 18 months after surgery, the patient is healthy. His creatinine level is 1.8 mg/dL, his erythrocyte sedimentation rate is 17 mm/h, and his C-reactive protein level is 7.71 mg/L.

## 3. Discussion

IgG4-related disease (IgG4-RD) is a recent nosological entity defined as a chronic immune-mediated fibro-inflammatory condition [1] characterized by tumefactive, tissue-destructive lesions or by organ failure [2]. IgG4-RD potentially involves nearly every anatomic site [3, 4], occasionally including urologic structures, as has been described in some short series of patients and in isolated case reports [5]. Typical histopathologic features are lymphoplasmacytic infiltrate rich in IgG4 plasma cells, obliterative phlebitis, and storiform fibrosis, while laboratory tests may reveal an inconstantly elevated serum IgG4 concentration [6].

In 1995, the concept of autoimmune pancreatitis (AIP) was proposed by Yoshida et al. [14]; in 2001, AIP type I was associated with high IgG4 serum levels [15]; and in 2003, IgG4-RD was described as the basic systemic condition responsible for AIP type I and for extrapancreatic lesions [16]. Since then, extrapancreatic manifestations of the disease have been increasingly reported in the literature [1, 4, 17].

As different terms have been proposed, the two independent teams headed by Umehara and Okazaki agreed to define the disease as IgG4-RD [18]. The disease can be difficult to diagnose because of a lack of confidence on the part of clinicians, pathologists, and radiologists [3] and because of its varying presentation [19].

Many papers have investigated urologic involvement [5], with the kidney being the most studied organ [5]. The first case of IgG4-RKD was described in 2004 [20, 21]. Serum IgG4 levels above 135 mg/dL [18] represent an important flag in the preliminary evaluation but do not represent either a necessary or sufficient condition for diagnosis [2]. However, high IgG4 serum levels seem to be related to the disease extension [2].

Our patient presented with normal IgG4 serum levels, sampled one month after surgery.

However, his IgG4/IgG ratio upon histopathologic examination was > 40%, which is considered a diagnostic criterion of IgG4-RKD [6].

IgG4-RKD is the most common among urologic manifestations of IgG4-RD, usually presenting in the form of TIN [7–9], although some cases of MGN have been described [9–11]. The diagnostic algorithm proposed by Kawano et al. [13] is shown in Table 1, and the algorithm by Raissian et al. [10] is summarized in Table 2.

In 2011, Kawano et al. described a series of 41 patients identified between 2004 and 2011 in Japanese hospitals presenting with histopathologic findings consistent with IgG4-RKD [13]. CT was performed for 29 patients; the most common radiologic findings were multiple low-density lesions, with other less frequent signs being diffuse bilateral renal swelling and diffuse thickening of the renal pelvis. A solitary hypovascular parenchymal nodule was detected in just one patient in this study; another patient probably had a unilateral renal mass causing renal swelling, but contrast-enhanced CT was not feasible because of decreased renal function. Of interest in the case we report, IgG4-RKD presented as a solid mass, thus mimicking malignancy. This is important because a differential diagnosis between IgG4-RKD and a renal neoplastic lesion is challenging.

It has been reported that conventional imaging—including ultrasound scans, CT, and MRI—has proven to be of limited usefulness in determining IgG4-RD [12].

Over the past decades, the management of overall renal masses has evolved from radical surgery to minimally invasive approaches, or even active surveillance in selected cases. Thus, the use of renal biopsy has shown an increase, although with a lack of standardization. Consequently, the identification of patients who may benefit from renal biopsy still remains an individualized clinical decision [22]. Nevertheless, renal biopsy represents a mainstay in the diagnosis of IgG4-RKD, including the differentiation between TIN and MGN [23, 24].

In our case, prior knowledge of the proper diagnosis could have directed the management towards medical therapy, thereby avoiding surgery at least as the first option (with the proviso that a biopsy would have given specific results that ruled out malignancy). That said, a smaller renal mass would definitely have been easier to manage for both diagnosis and treatment, because retrospective analysis could not establish whether our patient might have been an adequate responder to medical therapy due to the mass volume.

## 4. Conclusions

Concerning urologic sites, kidney is the organ most frequently involved in IgG4-RD.

However, the disease is rare and can be difficult to diagnose. Its typical clinical presentation includes altered renal function, with inconstant radiologic findings. In the case presented here, a single solid nodule was detected upon imaging evaluation, thus mimicking malignancy. This raises the issue of a proper preliminary differential diagnosis, which is not always easy. Conventional imaging—including ultrasound scans, CT, and MRI—has proven to be of limited usefulness in determining IgG4-RD. A multidisciplinary approach can be useful, although in clinical practice the selection of patients suspected of having IgG4-RKD is critical in cases presenting as a renal mass, thus mimicking malignancy.

## Figures and Tables

**Table 1:** Diagnostic criteria for IgG4-related kidney disease (IgG4-RKD) proposed by Kawano et al. [13].

(1) Presence of some kidney damage, as manifested by abnormal urinalysis or urine marker(s) or decreased kidney function with either elevated serum IgG level, hypocomplementemia, or elevated serum IgE level
(2) Abnormal renal radiologic findings:
(a) Multiple low-density lesions on enhanced computed tomography
(b) Diffuse kidney enlargement
(c) Hypovascular solitary mass in the kidney
(d) Hypertrophic lesion of renal pelvic wall without irregularity of the renal pelvic surface
(3) Elevated serum IgG4 level (IgG4 ≥ 135 mg/dl)
(4) Histologic findings in the kidney
(a) Dense lymphoplasmacytic infiltration with infiltrating IgG4-positive plasma cells > 10/high-power field (HPF) and/or IgG4/IgG-positive plasma cells > 40%
(b) Characteristics fibrosis surrounding nests of lymphocytes and/or plasma cells
(5) Histologic findings in extrarenal organ(s):
Dense lymphoplasmacytic infiltration with infiltrating IgG4-positive plasma cells > 10/HPF and/or IgG4/IgG-positive plasma cells > 40% in extrarenal organ(s)
Definite:
(1) + (3) + (4) (a), (b)
(2) + (3) + (4) (a), (b)
(2) + (3) + (5)
(1) + (3) + (4) (a) + (5)
Probable:
(1) + (4) (a), (b)
(2) + (4) (a), (b)
(2) + (5)
(3) + (4) (a), (b)
Possible:
(1) + (3)
(2) + (3)
(1) + (4) (a)
(2) + (4) (a)
Appendix:
(1) Clinically and histologically, the following diseases should be excluded: Wegener's granulomatosis, Churg-Strauss syndrome, and extramedullary plasmacytoma
(2) Radiologically, the following diseases should be excluded: malignant lymphoma, urinary tract carcinomas, renal infarction, and pyelonephritis (rarely, Wegener's granulomatosis, sarcoidosis, and metastatic carcinoma)
(3) Cases with suspected disease according to the diagnostic algorithm are classified into probable or possible IgG4-RKD according to these criteria

**Table 2:** Diagnostic criteria for IgG4-related tubulointerstitial nephritis (TIN) proposed by Raissian et al. [10].

Histology	Plasma cell-rich tubulointerstitial nephritis with >10 IgG4+ plasma cells/HPF in the most concentrated field^a^ Tubular basement membrane immune complex deposits by immunofluorescence, immunohistochemistry, and/or electron microscopy^b^

Imaging	Small peripheral low-attenuation cortical nodules, round or wedge-shaped lesions, or diffuse patchy involvement Diffuse marked enlargement of kidneys

Serology	Elevated serum IgG4 or total IgG level

Other organs involvement	Including autoimmune pancreatitis, sclerosing cholangitis, inflammatory masses in any organ, sialadenitis, inflammatory aortic aneurysm, lung involvement, and retroperitoneal fibrosis

Diagnosis of IgG4-TIN requires the histologic feature of plasma cell-rich TIN with increased IgG4+ plasma cells and at least one other feature from the categories of “imaging,” “serology,” or “other organ involvement”; ^a^mandatory criterion; ^b^supportive criterion, present in >80% of cases.
